# Relationships between narcissistic grandiosity, narcissistic vulnerability, regulatory focus, regulatory mode, and life-satisfaction: Data from two surveys

**DOI:** 10.1016/j.dib.2018.10.042

**Published:** 2018-10-18

**Authors:** Stephanie Hanke, Elke Rohmann, Jens Förster

**Affiliations:** Department of Psychology, Ruhr University Bochum, 44780 Bochum, Germany

## Abstract

The present data is reported in the article “Regulatory Focus and Regulatory Mode – Keys to Narcissists’ (Lack of) Life Satisfaction?” (Hanke et al., in press) [1]. The two data sets represent answers from two German samples. Data were collected via self-report questionnaires using EFS survey from QuestBack Unipark. The surveys included self-questionnaires of narcissistic grandiosity, narcissistic vulnerability, regulatory focus, regulatory mode, self-esteem, life-satisfaction, and demographic information.

**Specifications table**

**Table:** 

Subject area	*Psychology*
More specific subject area	*Social Psychology*
Type of data	*Quantitative data; Tables*
How data was acquired	*Online surveys using EFS survey from QuestBack Unipark*
Data format	*Analyzed data*
Experimental factors	*Correlational design*
Experimental features	*The main variables are narcissistic grandiosity, narcissistic vulnerability, regulatory focus, regulatory mode, self-esteem, and life-satisfaction*
Data source location	*Germany*
Data accessibility	*Data is with the article*
Related research article	S. Hanke, E. Rohmann and J. Förster, Regulatory focus and regulatory mode – keys to narcissists׳ (lack of) life satisfaction?, Pers. Indiv. Differ. 2018, https://doi.org/10.1016/j.paid.2018.09.039, (in press). [1]

**Value of the data**

• Raw data based on two data sets (*N*s = 297, 143) assessing narcissistic grandiosity, narcissistic vulnerability, regulatory focus, regulatory mode, self-esteem, life-satisfaction, and demographic information are provided.
• The data sets may be examined using a number of statistical methods such as analysis of variance, regression analysis, and structural equitation modeling.
• By reusing these data sets, researchers interested in narcissism and self-regulation may compare their own results with the data found in these two data sets.
• The data provided gives an insight into the relationships between grandiose and vulnerable narcissism, self-regulation, and life-satisfaction.

## 1 Data

Data set 1 contains self-report responses of 297 participants, data set 2 those of 143 participants. Table 1 lists the variables contained in data sets 1 and 2.

**Table 1:** Variables contained in data sets 1 and 2.

Variable name	Variable label	Variable values	Variable type
Gender	Gender	1 = male	Numeric
		2 = female	
Age	Age		Numeric
main_occ	Main occupation	1 = college student	Numeric
		2 = employed	
		3 = carer/homemaker	
		4 = apprentice	
		5 = unemployed	
		6 = retiree	
		7 = other	
Marital	Marital status	1 = single (never married)	Numeric
		2 = married	
		3 = divorced/separated	
		4 = widowed	
s_npi	Sum narcissistic grandiosity		Numeric
m_nir	Mean narcissistic vulnerability		Numeric
m_prom	Mean promotion focus		Numeric
m_prev	Mean prevention focus		Numeric
m_swls	Mean life satisfaction		Numeric
m_rses	Mean self-esteem		Numeric
Provided in data set 2 only:			
m_loco	Mean locomotion strength		Numeric
m_assess	Mean assessment strength		Numeric

## 2 Experimental design

The two data sets represent answers from two German samples. Data were collected using EFS survey from QuestBack Unipark. The two online questionnaires included self-report questionnaires of grandiose narcissism, vulnerable narcissism, regulatory focus, regulatory mode (provided in data set 2 only), self-esteem, life-satisfaction, and demographic information.

## 3 Materials and methods

Data set 1 (*N* = 297) was collected among university students at a large university in Western Germany and white-collar workers with an age range between 18 and 60 years (66 males, 231 females).

Data set 2 (*N* = 143) was also collected among university students at a large university in Western Germany and white-collar workers. The age ranged between 16–53 years (40 males, 103 females).

The following validated scales were used to assess the study variables (see Table 2):

**Table 2:** Overview of the measures utilized in data sets 1 and 2.

Study variable	Measure used	Original scale	German scale
Narcissistic grandiosity	Narcissistic Personality Inventory (NPI)	Raskin and Terry [2]	Schütz et al. [3]
Narcissistic vulnerability	Narcissistic Inventory-Revised (NI-R)	Deneke and Hilgenstock [4]	Rohmann et al. [5]
Promotion focus & Prevention focus	Regulatory Focus Questionnaire (RFQ)	Higgins et al. [6]	
Self-esteem	Rosenberg Self-Esteem Scale (RSES)	Rosenberg [7]	von Collani and Herzberg [8]
Life satisfaction	Satisfaction with Life Scale (SWLS)	Diener et al. [9]	Schumacher [10]
Locomotion & Assessment	Regulatory Mode Questionnaire (RMQ)	Kruglanski et al. [11]	Sellin et al. [12]

The NPI includes 40 forced-choice items, each consisting of two statements, which contrast a narcissistic alternative with a non-narcissistic alternative. The number of narcissistic alternatives chosen is calculated so that the total score of the NPI ranges from 0 to 40 with higher scores indicating higher grandiose narcissism. Example statements include “I am more capable than other people.” (narcissistic alternative) and “There is a lot that I can learn from other people”.

The NI-R consists of 36 items. Participants respond on five-point Likert scales (1 = *not at all true*; 5 = *completely true*). The mean across scale items is calculated with higher scores indicating higher vulnerable narcissism. Items include statements such as “I would really enjoy being praised for everything I do (like a child is praised by its parents)”.

The RFQ contains two subscales: The promotion subscale encompasses six items and assesses individuals׳ subjective histories of promotion success whereas the prevention subscale contains five items and assesses individuals׳ subjective histories of prevention success. Participants respond on five-point Likert scales (1 = *not at all true*; 5 = *completely true*). The mean across scale items is calculated with higher scores on either subscale reflecting individuals׳ sense of their history of promotion or prevention success in goal attainment, respectively. Sample items would be “How often have you accomplished things that got you ׳psyched׳ to work even harder?” (promotion focus) and “How often did you obey rules and regulations that were established by your parents?” (prevention focus).

The RSES is a widely used and validated self-report instrument for measuring explicit and global self-esteem by assessing positive and negative feelings about the self. The RSES consists of 10 items which are rated on a 4-point Likert scale format ranging from 1 (*strongly agree*) to 4 (*strongly disagree*). A sample item would be “On the whole, I am satisfied with myself”.

The SWLS is a five-item scale designed to measure global cognitive judgments of satisfaction with one׳s life. The items are rated on seven-point scales ranging from 1 (*strongly disagree*) to 7 (*strongly agree*). Items include statements such as “I am satisfied with my life”.

The Regulatory Mode Questionnaire assesses locomotion with 12 items and assessment with 10 items. The items are rated on six-point Likert scales ranging from 1 (*strongly disagree*) to 6 (*strongly agree*). Example items are: “When I decide to do something, I can׳t wait to get started.” (locomotion) and “I often critique work done by myself or others.” (assessment).

Descriptive statistics (means, standard deviations, Cronbach׳s alpha) were computed for each measure and can be found in Table 3, Table 4. In the case of the NPI, summary scores were computed instead of means.

**Table 3:** Descriptive statistics and gender differences of narcissism, regulatory focus, and life satisfaction (data set 1).

Scale	Total sample (*N* = 297)			Men (*N* = 66)		Women (*N* = 231)		*F*	ƞ^2^	Skewness	Kurtosis
	Alpha	*M*	*SD*	*M*	*SD*	*M*	*SD*				
Grandiosity^a^	0.82	13.91	6.31	16.65	7.45	13.14	5.73	16.77***	0.50	0.57	0.06
Vulnerability^c^	0.85	2.74	0.47	2.76	0.48	2.74	0.47	0.12	0.00	0.02	− 0.20
Self-esteem^b^	0.90	3.11	0.68	3.23	0.60	3.08	0.69	2.46	0.01	− 0.75	− 0.13
Promotion^c^	0.64	3.54	0.64	3.59	0.65	3.53	0.64	0.40	0.001	− 0.25	− 0.11
Prevention^c^	0.73	3.32	0.75	3.24	0.79	3.35	0.74	1.09	0.004	− 0.28	− 0.09
Life satisfaction^d^	0.87	4.95	1.23	4.83	1.34	4.99	1.19	0.86	0.003	− 0.69	− 0.12

*Note. N* = 297. SWLS = Satisfaction with Life Scale.

a Values range from 0 to 40.

b 4-point Likert-type scale.

c 5-point Likert-type scale.

d 7-point Likert-type scale.

*** *p* <.001.

**Table 4:** Descriptive statistics and gender differences of narcissism, regulatory focus, regulatory mode and life satisfaction (data set 2).

Scale	Total sample (*N* = 143)			Men (*N* = 40)		Women (*N* = 103)		*F*	ƞ^2^	Skewness	Kurtosis
	Alpha	*M*	*SD*	*M*	*SD*	*M*	*SD*				
Grandiosity^a^	0.85	13.70	6.68	13.48	7.47	13.78	6.39	0.06	0.00	0.72	0.48
Vulnerability^c^	0.87	2.68	0.48	2.73	0.49	2.67	0.48	0.50	0.004	0.29	0.03
Self-esteem^b^	0.87	3.25	0.56	3.30	0.50	3.23	0.58	0.44	0.003	− 0.75	0.14
Promotion^c^	0.63	3.63	0.61	3.61	0.67	3.64	0.60	0.07	0.00	− 0.36	- 0.12
Prevention^c^	0.76	3.35	0.81	3.22	0.76	3.40	0.83	1.56	0.01	− 0.56	0.23
Life satisfaction^e^	0.87	5.13	1.22	5.10	1.10	5.15	1.27	0.06	0.00	− 1.04	1.14
Locomotion^d^	0.74	4.17	0.57	4.09	0.58	4.20	0.56	0.95	0.01	− 0.45	0.11
Assessment^d^	0.65	4.02	0.61	3.89	0.60	4.07	0.61	0.11	0.02	0.27	− 0.69

*Note. N* = 143. SWLS = Satisfaction with Life Scale.

a Values range from 0 to 40.

b 4-point Likert-type scale.

c 5-point Likert-type scale.

d 6-point Likert-type scale.

e 7-point Likert-type scale.

Further, we tested for gender differences in the study variables through an ANOVA (see Table 3, Table 4). In data set 1, men (*M* = 16.65, *SD* = 7.45) expressed more narcissistic grandiosity than women (*M* = 13.14, *SD* = 5.73), *F*(1, 290) = 16.77, *p* < .001, ƞ^2^ = 0.05. In data set 2, tests for gender differences turned out to be not significant.
