# Supplementary material for: Relationships between narcissistic grandiosity, narcissistic vulnerability, regulatory focus, regulatory mode, and life-satisfaction: Data from two surveys
Source: Data Brief. 2018 Oct 18;21:861–5. doi: 10.1016/j.dib.2018.10.042 (PMC6223186; doi:10.1016/j.dib.2018.10.042)
Supplement: Supplementary file 1 — Transparency document. [file mmc1.docx]

**Conflict of Interest Form**

We wish to confirm that there are no known conflicts of interest associated with this publication and there has been no significant financial support for this work that could have influenced its outcome.

Stephanie Hanke, Elke Rohmann & Jens Förster
